# Do Children with High Callous-Unemotional Traits Have Attentional Deficits to Emotional Stimuli? Evidence from a Multi-Method and Multi-Informant Study

**DOI:** 10.1007/s10578-024-01739-6

**Published:** 2024-08-17

**Authors:** Anastasiya Ivanova-Serokhvostova, Kostas Fanti, Albert Bonillo, Hans Supèr, Montserrat Corrales, Iris Pérez-Bonaventura, Montserrat Pamias, Antoni J. Ramos-Quiroga, Rafael Torrubia, Roser Nadal, Paul J. Frick, Beatriz Molinuevo

**Affiliations:** 1 Department of Psychiatry and Forensic Medicine, Universitat Autònoma de Barcelona (UAB), Cerdanyola del Vallès, Spain; 2 Institut de Neurociències, Universitat Autònoma de Barcelona, Cerdanyola del Vallès, Spain; 3 Department of Psychology, University of Cyprus, Nicosia, Cyprus; 4 Department of Psychobiology and Methodology of Health Sciences, Universitat Autònoma de Barcelona, Cerdanyola del Vallès, Spain; 5 Catalan Institution for Research and Advanced Studies (ICREA), Barcelona, Spain; 6 Institute of Neurosciences, Department of Cognition, Development and Education Psychology, Universitat de Barcelona, Barcelona, Spain; 7 Department of Psychiatry, Hospital Universitari Vall d’Hebron, Barcelona, Spain; 8 Group of Psychiatry Mental Health and Addictions, Vall d’Hebron Research Institute (VHIR), Barcelona, Spain; 9 Department of Mental Health, Hospital Universitari Parc Taulí, Sabadell, Spain; 10 Centro de Investigación Biomédica en Red de Salud Mental (CIBERSAM), Madrid, Spain; 11 Department of Clinical and Health Psychology, Universitat Autònoma de Barcelona, Cerdanyola del Vallès, Spain; 12 Department of Psychology, Louisiana State University, Baton Rouge, USA

**Keywords:** Callous-unemotional, Emotion recognition, Visual behavior, Eye gaze, Children, Adolescents

## Abstract

Callous-unemotional (CU) traits in children and adolescents are linked to severe and persistent antisocial behavior. Based on past empirical research, several theoretical models have suggested that CU traits may be partly explained by difficulties in correctly identifying others’ emotional states as well as their reduced attention to others’ eyes, which could be important for both causal theory and treatment. This study tested the relationships among CU traits, emotion recognition of facial expressions and visual behavior in a sample of 52 boys referred to a clinic for conduct problems (*M*age = 10.29 years; *SD* = 2.06). We conducted a multi-method and multi-informant assessment of CU traits through the Child Problematic Traits Inventory (CPTI), the Inventory of Callous-Unemotional (ICU), and the Clinical Assessment of Prosocial Emotions-Version 1.1 (CAPE). The primary goal of the study was to compare the utility of these methods for forming subgroups of youth that differ in their emotional processing abilities. An emotion recognition task assessed recognition accuracy (percentage of mistakes) and absolute dwell time on the eyes or mouth region for each emotion. Results from repeated measures ANOVAs revealed that low and high CU groups did not differ in emotion recognition accuracy, irrespective of the method of assessing CU traits. However, the high CU group showed reduced attention to the eyes of fearful and sad facial expressions (using the CPTI) or to all emotions (using the CAPE). The high CU group also showed a general increase in attention to the mouth area, but only when assessed by the CAPE. These findings provide evidence to support abnormalities in how those elevated on CU traits process emotional stimuli, especially when assessed by a clinical interview, which could guide appropriate assessment and more successful interventions for this group of youth.

## Introduction

Callous-unemotional (CU) traits are characterized by lack of remorse and guilt, a callous lack of empathy, shallow affect, and limited concern about performance in important activities [1]. CU traits can be found in children without significant conduct problems, in whom they still predict impairment [2–4]. However, they have been most commonly used in research to identify a subgroup of youth with serious conduct problems who show a more severe, stable, and aggressive pattern of antisocial behavior starting in early childhood [1] and who are less responsive to many traditional mental health treatments [5]. As a result of this evidence for their clinical utility, CU traits have been included in mental health diagnoses as a Limited Prosocial Emotions (LPE) specifier for Conduct Disorder (CD) in the 5th edition of the Diagnostic and Statistical Manual (DSM-5; American Psychiatric Association [APA], [6]) and for both CD and Oppositional Defiant Disorder (ODD) in the 11th edition of the International Classification of Diseases (ICD-11; World Health Organization [WHO], [7]).

CU traits have been associated with the affective features of psychopathy [8, 9] and with a greater risk for showing antisocial and psychopathic traits in adulthood [10]. Additionally, CU traits are correlated with other personality dimensions that have also been associated with psychopathic traits in adults (e.g., grandiosity, deceitfulness, irresponsibility, or impulsivity [11]), many of which are included in definitions of childhood disruptive behavior disorders [e.g., impulsivity in the criteria for Attention-Deficit/Hyperactivity Disorder (ADHD) or deceitfulness in the criteria for Conduct Disorder (CD) [12]]. However, despite being correlated with other personality dimensions, CU traits are often uniquely associated with emotional deficits [13–15] and are most useful for designating subgroups of youth with conduct problems who differ in their emotional deficits [12]. These emotional differences have been critical for informing both etiological theories for how conduct problems develop in the various subgroups of children who differ on their level of CU traits (see [1, 16] for reviews) and for informing how to tailor treatment for the different subgroups of children with conduct problems [17]. In terms of causal theories, the ability to recognize emotions and differentiate among the different emotions has been considered necessary for a child to develop an awareness of social cues and to respond appropriately to normal socialization processes [18]. Accurate face recognition involves understanding others’ thoughts, feelings and intentions, which then can elicit empathic concern towards others and promote prosocial behavior [19]. Even more importantly for understanding their potential role in the development of conduct problems, impaired emotional recognition [20] and insensitivity to distress cues in others, such as fear and sadness, can disrupt the mechanism that usually prevents people from committing behaviors that harm others [21–23]. Consistent with these theories, individuals high on CU traits, which are defined by empathy deficits, have been shown to have difficulties in recognizing facial expressions of emotions in others [20, 24–26], especially fearful and sad facial expressions [27–31]. These findings were recently supported in a meta-analysis [32]. It is important to note that some research has failed to link inaccurate recognition with CU traits [30, 33, 34]. However, even when no differences in accuracy were found for those elevated on CU traits, some problems in emotional processing in those elevated on CU traits were reported, such as CU traits being related to a slower processing speed when identifying angry, sad, and fearful faces [34].

Given the potential importance of problems associated with recognizing emotions in others in the development of CU traits, research has begun to focus on more basic emotional processes that might underlie these deficits. For example, early deficits in a child’s emotional reactivity to certain types of social cues, such as distress in others, may reduce a child’s motivation for learning to recognize others’ emotions, since they do not experience the aversive emotional arousal that motivates such learning in those without the deficits in emotional reactivity [18, 35]. Another theoretical approach by Dadds and colleagues [28, 29, 36] proposes that the identified emotion recognition deficits might be due to a reduced attention to socially relevant cues in the faces of others, such as reduced orienting to the eye region of faces. Differences in eye-gaze can predict someone’s ability to capture others’ intentions, as well as the meaning of social situations [37]. However, the most atypical processing may be specifically related to fearful expressions, since the eyes are the most important area for identifying this emotion [38]. This possibility has been supported by brain imaging research, which has suggested that the amygdala is an important brain structure for attentional orienting to fearful and sad facial expressions and which has suggested that reduced amygdala response to fearful and sad faces is often found in youth with conduct problems who are also elevated on CU traits [39, 40].

Based on this theory, one would predict that a deficit in the attention to the eye region would be found in children elevated on CU traits. However, several recent eye-tracking studies have reported inconclusive findings. In support of this deficit, studies of children with conduct problems from community and clinical samples found that CU traits were related to reduced attention to others’ eyes, whereby CU traits were associated with fewer and shorter fixations [25, 27, 28] or with a lower likelihood to fixate first on the eye region of facial stimuli [28, 30]. Importantly, these deficits seemed to be specifically due to deficits in attention to the eye region, since either no attentional deficits to the mouth area were observed [28] or even enhanced attention to the mouth region was reported [25]. Furthermore, Dadds et al. [28, 41] concluded that the attentional preference to the eye region was key in the emotion recognition deficits of children with elevated CU traits, given that when children were instructed to look at the eyes of the facial stimuli, the deficits in emotion recognition linked to CU traits disappeared and then reappeared when they were instructed to focus on the mouth area.

While these findings are important for potentially uncovering the emotional processes leading to the deficits in emotional recognition experienced by children with elevated CU traits, such attentional deficits have not always been consistently found [26, 34, 42, 43]. Also, like the findings on emotional recognition, there is still debate about whether the deficits in eye gaze are related to specific emotions or to emotional processing more generally. Some studies report that children with elevated CU traits show reduced attention to others’ eyes for a large number of emotions [25, 28], with more pronounced deficits in paying attention to fearful expressions [28]. Other studies reported reduced attention only to sad [27] or surprised facial expressions [30] among individuals high on CU traits.

## The Current Study

In sum, while deficits in the ability to recognize others’ emotions have been critical to many theories for how CU traits develop, there are still some important unanswered questions that could be critical for both causal theory and for designing more effective treatment for children with conduct problems and elevated CU traits. For example, it is still unclear how broad (i.e., to many emotions) or specific (e.g., to fear or distress only) these deficits might be and whether or not they could be explained by a child’s attention to certain regions of the face, such as reduced attention to the eye region. Even more importantly, there are inconsistencies in findings across studies. While many studies have found that CU traits often show reduced accuracy in recognizing emotions in others’ faces and often do not attend to the eye regions in the faces of others, many studies have not found these associations with CU traits.

One possible explanation for these inconsistencies in findings across studies that has not been tested to date is whether they are related to the method for assessing CU traits. The method for assessing CU traits is critical, given that the subgroups of children with conduct problems who differ on their level of CU traits show very different patterns of emotional deficits. That is, it may not be the case that the subgroup with elevated CU traits shows emotional deficits and those without elevations in CU traits show more normative patterns of emotional functioning. Instead, both groups may show emotional deficits, but the deficits may differ across groups. As a result, misclassifications of youth could have dramatic effects on the results of studies. To illustrate this, Viding et al. [44] studied functional MRI responses to fearful and calm faces in a sample of boys (ages 10–16) and reported that amygdala responses to fearful faces (relative to calm faces) were stronger than controls in boys with conduct problems without elevated CU traits but were weaker compared to controls in boys with conduct problems who were elevated on CU traits. Similarly, in a sample of children (*M*age = 11.21; *SD* = 1.06), Fanti et al. [45] reported that on both physiological (i.e., startle reflex during fear imagery) and behavioral (i.e., ratings of fear and sensitivity to punishment) measures of fearfulness, children with chronic conduct problems with and without CU traits showed opposing responses, with those high on CU traits showing weaker startle reflex and lower ratings of fear and punishment sensitivity and those normative on CU traits showing enhanced physiological responses and higher behavioral ratings. While these studies did not specifically study either emotional recognition or attention to the eye regions in human faces, they do illustrate the importance of accurately forming subgroups of youth with conduct problems, since misclassifications could lead to inconsistent findings.

To begin to address these limitations in past research, the current study investigated whether youth with elevated CU traits showed deficits in their ability to recognize others’ emotions, and in their visual attention to the faces of others showing emotions, using eye-tracking technology. We studied a sample of boys (6–14 years old) who were referred to mental health clinics for significant conduct problems. We limited our study to only boys, given the relatively low number of girls who are referred to the clinics for conduct problems. Based on past research, we predicted that boys with elevated CU traits would show more impairments in their ability to recognize emotions in the faces of others and would be less likely to pay attention to the eye region of these faces. Further, based on past research, we hypothesized that boys high on CU traits would show increased gaze towards the eyes when instructed to pay attention to the eyes of the faces, which would improve their emotion recognition accuracy. Finally, based on past research, we predicted that this deficit would be present even after controlling for other personality dimensions (i.e., grandiosity, deceitfulness, impulsivity, need for stimulation) that have been found to be related to conduct problems in past samples.

Most importantly, we used a methodology that could help to explain some of the inconsistent findings in past research. First, we determined whether or not any deficits in emotional recognition or attentional orienting to faces shown by those high on CU traits were found across emotions or were specific to certain emotions (i.e., fear and sadness). Second, we compared findings across groups that were formed through different methods. That is, we compared groups elevated on CU traits, as defined by the use of behavior rating scales; namely, the Child Problematic Traits Inventory (CPTI; [46]) and the Inventory of Callous-Unemotional Traits (ICU; [47]). This methodology replicates how groups have typically been formed in past research. However, we compared our findings using this common approach to group formation with findings using a more extensive assessment that was designed to more rigorously differentiate those with and without normative levels of CU traits. That is, the Clinical Assessment of Prosocial Emotions. Version 1.1 (CAPE; [48]) that uses professional clinical judgment that attempts to weigh information obtained across multiple informants to determine if a child meets the criteria for the diagnosis of the LPE specifier. Importantly, the CAPE is unique in directly corresponding to the diagnostic criteria included in the DSM-5. Further, the CAPE allows for a comprehensive clinical assessment of not only whether the child shows CU traits but how impairing, persistent and pervasive these traits are. Thus, given the more comprehensive nature of the CAPE and its design to specifically place children into diagnostic subtypes, we tested the novel prediction that deficits in emotion recognition and attentional deficits to facial regions would be more pronounced in groups diagnosed with the LPE specifier based on CAPE, in comparison to groups formed using elevations on the CPTI or ICU rating scales.

## Method

### Participants

The sample was composed of children and early adolescents participating in a research project aimed at studying emotional and attentional correlates of conduct problems (EMPROLIMIT study). A total of 52 boys aged 6–14 years (*M* = 10.29 years, *SD* = 2.06) who were referred to outpatient clinics at two hospitals in the area of Barcelona (Spain) [Vall d’Hebron Hospital (*n* = 30; 58%) and Parc Taulí Hospital (*n* = 22; 42%)] for the assessment and treatment of conduct problems participated in the present study. The inclusion criteria were: a) to identify as male; b) absence of significant intellectual deficits; c) absence of explicit difficulties in speaking and understanding the Spanish language; d) absence of schizophrenia, psychotic disorders, autism spectrum disorder symptoms, and significant sensory or motor deficits; and e) available assessment of CU traits, based on information from parents and teachers. Intelligence was screened using the Vocabulary and Matrix scale scores of the Wechsler Intelligence Scale for Children – Fourth Edition (WISC-IV; [49]). The two-subtest screening was used because of its strong associations with a total IQ score: Vocabulary (*r* = 0.73) and Matrix Reasoning (*r* = 0.63) [49]. Scores greater than 4 (less than two SDs below the median of the scale scores *M* = 10, *SD* = 3) were required for inclusion. The *Mini International Neuropsychiatric Interview for Children and Adolescents for DSM-5* (MINI-KID 7.0.2; [50]) was used to screen for co-morbid forms of psychopathology and to assess for inclusion criteria for most participants (5 clinical referrals were included based on other clinical assessments). The MINI-KID was administered to children/adolescents in the presence of their parents.

Participants were predominantly Caucasian (88%), followed by Latinos (8%), and others (4%). Parents were most commonly university/college graduates (50% mothers, 25% fathers) or high school graduates (29% mothers, 31% fathers). Diagnostic characteristics of the participants were classified as externalizing disorders (at least one of the following diagnoses: CD, ODD, or ADHD), internalizing disorders (at least one: major depression, suicidal behavior, specific phobia, social phobia, obsessive–compulsive disorder, separation anxiety, or generalized anxiety disorder), and tic disorders according to MINI-KID 7.0.2 (*n* = 47; [50]) or *Kiddie-Schedule for Affective Disorders and Schizophrenia-Present and Lifetime version* (*n* = 5; [51]). Twenty-four participants (46%) showed externalizing disorders; two participants (4%) presented internalizing disorders; six participants (11%) met criteria for both internalizing and externalizing disorders; three participants (6%) had externalizing and tic disorders; one participant showed externalizing, internalizing and tic disorders (2%); and sixteen (31%) participants did not meet criteria for any disorder, despite being referred for showing significant conduct problems.

### Measures (CU Traits)

*Child Problematic Traits Inventory (CPTI;* [46]); authorized Spanish version [52]. Parents and teachers (*n* = 52) rated the 28 items of the CPTI on a 4-point Likert scale ranging from 1 (*Does not apply at all*) to 4 (*Applies very well*), based on how the child usually behaves. To avoid underreporting by an informant and to incorporate information about different settings, scores from parents and teachers were combined by using the higher score from either informant for each item. This method has been recommended for combining informants on CU traits, since a) there is more motivation for persons to underreport than over-report symptoms and b) those who may be able to hide the traits in certain settings (i.e., score higher in one setting than another) may actually show more severe levels of the traits [53, 54].

The 28 items not only assess CU traits (10 items), but they also assess other traits that were used as control variables in analyses: Grandiose- Deceitful (GD; 8 items) and Impulsive-Need for Stimulation (INS; 10 items). In the current study, Cronbach’s alphas of the parents (teachers) version were 0.88 (0.92), 0.91 (0.94), and 0.85 (0.86) for the GD, CU-CPTI, and INS traits, respectively. The correlations between parents’ and teachers’ reports were small and significant only for INS: GD (*r* = 0.12, *p* = 0.40), CU-CPTI (*r* = 0.10, *p* = 0.48), INS (*r* = 0.29, *p* = 0.04). The median split on CU-CPTI scale was used to create low (*n* = 27) and high (*n* = 25) groups on CU traits.

*The Inventory of Callous-Unemotional Traits (ICU*, [47]); authorized Spanish version [55]*.* Parents and teachers (*n* = 50) rated the 24-items of the ICU on a 4-point Likert scale ranging from 0 (*Not at all true*) to 3 (*Definitely true*) about the child. As with the CPTI, the highest score on each item from either informant was used to determine a total scale score. In the current study, Cronbach’s alphas of the ICU total score for the parents’ and teachers’ versions were 0.84 and 0.92 respectively. The correlation between parents’ and teachers’ reports was modest but significant (*r* = 0.28, *p* = 0.05). The median split on ICU total score was used to create low (*n* = 25) and high (*n* = 25) groups on CU traits.

*Clinical Assessment of Prosocial Emotions. Version 1.1 (CAPE;* [48], Spanish and Catalan versions [56]). The CAPE is a clinician rating designed specifically to assess the DSM-5 LPE specifier for the diagnostic criteria for CD in persons from ages 3 to 21. The four criteria indicated by the DSM-5 are evaluated: (a) lack of remorse or guilt; (b) callous-lack of empathy; (c) unconcerned about performance; (d) shallow or deficient affect. It uses the structured professional judgment method, where prototypes for each key indicator of CU traits are provided in order to guide the clinician using the tool. It is designed to be used by clinicians with experience and knowledge of the assessment of childhood psychopathology in general and of CU traits specifically. In this study, the raters were members of the research team with experience in childhood psychopathology. All raters received individual and group training sessions following the training guidelines provided for the CAPE [48]. Training sessions were conducted by the member of the research team (B.M.) who in turn had received training from the CAPE’s creator (P.J.F.).

Clinical judgments were based on semi-structured interviews with multiple informants [parents and teachers (*n* = 51) and participants who were older than 10 were also interviewed (*n* = 34)]. Other sources of information (e.g., records, observation) could also be used for scoring the CAPE. For the interviews with the informants, each item starts with a stem question (e.g., “Does ________ seem to feel bad or guilty if he/she does something wrong or if he/she hurts someone?”) that must be answered as either “yes” or “no” by the informant. The stem questions are followed by a request for examples during which the clinician can ask whatever follow-up questions they feel are indicated to obtain enough information to make ratings of the four symptoms. The LPE specifier is considered as present when at least two symptoms are considered by the clinician to be “Highly descriptive” of the child. Two groups were created based on the presence (*n* = 13) or absence (*n* = 38) of the LPE specifier. The interviews were recorded (audio) and scored by two raters.^1^ The interrater reliability of the specifier was substantial (*n* = 44; *k* = 0.69, *p* < 0.001) and is consistent with the reliability reported in other samples using the CAPE [57, 58]. Importantly, Goetz et al. [57] showed that diagnoses of the LPE based on the CAPE led to subgroups of clinic-referred children and adolescents with serious conduct problems who differed in the severity of their conduct problems.

### Material (Emotional Task)

#### Facial Expression Stimuli

Three different adult male models were selected from the NimStim Face Stimulus Set [59] and another three adult male models were selected from the Pictures of Facial Affect (POFA; [60]). A different male model was selected for the training pictures [59]. Faces were selected based on the highest mean proportion of correct answers for the chosen expressions. The models conveyed happiness, sadness, anger, disgust, fear and a neutral expression. Each image was converted to grayscale and an elliptic mask was applied in order to reveal only the face without hair and ears. The pictures had a size of 506 × 650 pixels.

#### Adaptation of the University of New South Wales Facial Emotion Task

To measure both accuracy of emotion recognition and visual behavior in response to emotional faces, we used an adaptation of the University of New South Wales Facial Emotion Task (UNSW FACES; [28, 29]). A PowerPoint presentation was created in which happiness, sadness, anger, disgust, fear, or a neutral expression was each displayed by the six adult male static faces. The participants were told that slides with different emotional expressions would appear very quickly, and then they would have to choose from a list to indicate which emotion appeared. Since participants had to choose an emotion from the list before moving on to the next face, there were no missing data for this task.

The task consisted of four conditions (blocks) following this order: free gaze, eye gaze, mouth gaze and free gaze again. In block 1, all 6 emotions were presented 6 times (36 trials), then only two emotions appeared 6 times in blocks 2, 3 and 4 (12 trials each). Emotions were presented in a semi-randomized order, with the restriction that the same emotion was not presented more than twice consecutively in each block. In blocks 2 and 3, participants were asked to focus on eyes or mouth, respectively. To aid in following these instructions, a slide displaying an X was presented before each face slide, either in the eye or mouth region. To make sure that participants understood the task, an example of each expression was presented before the task and there were also two training trials before each block except for block 4, which included only one training trial. The block 4 free gaze was included to help individuals return to their natural gaze condition for ethical reasons but was excluded from the analyses.

Each trial started with a black screen for 2 s. In blocks 2 and 3, the X slide also appeared for 1 s after the black screen. Then a face slide was presented randomly for 2 s each. Finally, a list with all six emotions appeared on the screen, and the participants chose the one that they saw. The experimenter registered the answer and asked the participants to press a button in order to move on to the next trial. Participants were given as long as necessary to make their selection and were not given feedback about their performance accuracy. The total duration of the task was between 15–20 min, depending on how much time the participants needed to answer or understand the instructions. A correct response was scored 1 and an incorrect response was scored 0. The percentage of mistakes was computed for each emotion and block.

#### Eye Tracking Data

BGaze system (Braingaze SL, Mataró, Spain) was used for stimuli presentation synced with a portable Tobii X2-30 to register the eye movements (Tobii Technology, AB, Sweden). Tobii X2-30 is a compact binocular eye-tracker sampling at 30 Hz with a 22-inch display, with a maximum resolution of 1.024 × 768 pixels. The Tobii allows real-time tracking of both eyes and analysis of specific areas of interest (AOI). For the AOIs, in line with Dadds et al. [28], two regions were defined: a rectangle fitted to the eyes area, and a rectangle adjusted to the mouth area. The AOIs were fixed for all the stimuli and were defined to ensure that they included the eye and mouth areas of all the faces. Before the UNSW task, the equipment was calibrated for each participant using a standard procedure with five fixation points at the beginning of the experiment. The quality of calibration was accurate with at least 1000 ms of gaze fixation to collect 30 gaze points at each calibration location within a radius of 50 pixels from the center of the point. The children’s eyes were positioned at 55 to 60 cm from a computer monitor. To minimize movements, participants were supported by a chinrest and were seated in a fixed chair.

From the eye tracker output data, gaze points and gaze point velocities were calculated. While the velocity was kept below a given threshold (0.05 units/Δt, Δt = ‘sampling interval’ = 33.33 ms), the movement was considered to be ‘fixational’. All the points connected by eye movements of that type were assigned to the same fixation. When the velocity became higher, the current fixation was ‘broken’, and the search for new fixations started again. Trials were excluded if there was no valid data for at least 25% of the trial duration, and children with less than 50% of valid trials were also excluded (*n* = 1). We computed the absolute dwell time (microseconds, μs) on the eyes or mouth region for each emotion and block representing general attention orienting during the whole stimuli presentation [25, 33]. For the analyses of blocks 2 and 3, the absolute dwell times were multiplied by three to make them comparable to block 1. The first fixation was disregarded to make the blocks comparable, since in blocks 2 and 3 the participants were asked to fixate their attention on a cross.

### Procedure

Selection of participants at each hospital consisted of a session in which the candidates were assessed for intelligence with the WISC-IV Matrix and Vocabulary subtests and either the MINI-KID (*n* = 47) or other clinical assessment (*n* = 5). The session lasted approximately 2 h. If the candidates fulfilled the criteria to be included in the study, they and their families were formally invited to participate in it. After the boys and their parents both agreed, they were referred to the Human Psychophysiology lab for a second session and the parents provided informed consent and the child assented to participation. All participants were instructed to refrain from using psychostimulant medication for at least 24 h before the experimental task. The second psychological assessment and the emotion recognition task were performed in two different locations and always required the presence of the participants and their families. The participants (children and parents) recruited at the Vall d’Hebron Hospital were tested in that hospital. The participants and their families recruited at the Parc Taulí Hospital were tested at the Human Psychophysiology lab located in the Medical Psychology Unit at the University Campus. The same equipment was used in both labs (eye-tracker and computers) and both places were quiet and dimly lit. The assessment session included a questionnaire developed by the research team to collect sociodemographic data, the CPTI and ICU completed by one of the parents, the CAPE interview, and other questionnaires, which were not used in the present study but are part of the larger data collection. Occasionally, this testing session had to be split in two to adjust the assessment to the families’ needs or the children’s characteristics. Within approximately one month,^2^ the teachers completed the CAPE by phone and CPTI, ICU and other questionnaires online. When scoring rating scales, if less than 30% of items of a scale had missing responses, then the total score of the scale was computed as a mean of the available items. However, in this study, we only identified 3 missing items from 3 different people in the parents’ version of the ICU. There were no missing data for the CPTI or CAPE responses. Also as noted, when the teacher’s questionnaire was missing, the participant was eliminated from the analyses (*n* = 2 for the ICU). The emotion recognition task for each child was conducted in parallel with the second assessment session for parents. A researcher (AI) who was blinded to the participants’ diagnosis carried out this part of the study. Upon arrival at the lab, the instructions for the experiment were read to the participants, and they were asked to pay attention to the screen and use the chin rest during the whole task.

### Data Analyses

First, associations among the various measures of CU traits were tested. Second, we tested whether there were differences in emotion recognition and visual behavior between emotions and blocks. Emotion type and block (6 × 3) were included as within-subject factors in a repeated-measures ANOVAs (GLM procedure) and percentage of mistakes or total duration of fixations to eyes or mouth as dependent variables. Third, the statistical distribution of estimated values was graphically examined and approximated a normal distribution, without outliers. Importantly, repeated-measures ANOVAs are robust to non-normal distributions [61]. The sphericity assumption is the most important assumption for repeated-measures designs, and this was tested. When a violation of this assumption was evident, the Greenhouse–Geisser adjustment was used, which is indicated by an *F* value with decimals in the degrees of freedom. Fourth, we tested if CU traits were related to emotion recognition or visual behavior deficits. Emotion recognition accuracy and visual behavior data were analyzed using separate repeated-measures ANOVAs (GLM procedure) for each dependent variable (percentage of mistakes and total duration of fixations to eyes or mouth) and CU traits measure (CU-CPTI, ICU or CAPE). Emotion type and block were included as within-subject factors and group (low vs high CU traits or absent vs present LPE specifier) as a between-subjects factor. GD traits, INS traits, and age were used as covariates. When either main or interaction effects were significant, univariate follow-up Bonferroni tests were computed. Partial eta square ƞ^2^ (≥ 0.01 small, ≥ 0.06 medium, ≥ 0.14 large) are reported as a measure of the effect size for repeated measures ANOVA main and interaction effects. Cohen’s *d* (small ≥ 0.20, medium ≥ 0.50, large ≥ 0.80; [62]) is computed as a measure of the effect size for *T*-test analyses and follow-up Bonferroni tests. All analyses were conducted using IBM SPSS 22.0.

Finally, as this was a clinic-referred sample that was part of a broader data collection procedure, an a priori power analysis was not conducted. However, a post-hoc power analysis was computed using the G*Power program [63] for main effects and interactions. For this analysis, Type I error (α) was fixed at 0.05 and Type II (1-β) error at 0.20. The sample size was set to *n* = 50 and three covariates were considered. The power was sufficient to detect an effect size (Cohen’s *d*) of 0.58, which is considered a medium size effect.

## Results

### Associations Among Measures of CU Traits

Zero-order correlations showed that all three measures of CU traits were strongly correlated: CU-CPTI-ICU (*r* = 0.86, *p* < 0.001), ICU-CAPE (*r* = 0.69, *p* < 0.001), and CU-CPTI-CAPE (*r* = 0.70, *p* < 0.001). *T*-test results revealed that children with the LPE specifier according to the CAPE showed significantly higher scores on CU-CPTI (*M* = 2.96; *SD* = 0.55; *t*(49) = 6.27; *p* < 0.001; *d* = 2.06) and ICU (*M* = 1.97; *SD* = 0.44; *t*(47) = 4.15; *p* < 0.001; *d* = 1.41) scales than those without the LPE specifier (CU-CPTI: *M* = 1.89; *SD* = 0.53; ICU: *M* = 1.41; *SD* = 0.39).

### Emotion Recognition Accuracy

First, we analyzed the task performance in the whole sample (see Table 1 for descriptive statistics). During free gaze, the number of mistakes was low for most emotions, with the exception of sadness (50%), followed by disgust (27%) and anger (27%). Findings from the repeated-measures ANOVA for emotional recognition accuracy revealed significant effects of block, *F*(1.71, 88.77) = 40.35; *p* < 0.001; *ƞ*_*p*_^*2*^ = 0.44, and emotion *F*(3.83, 195.31) = 37.58; *p* < 0.001; *ƞ*_*p*_^*2*^ = 0.42. Also, a significant block*emotion effect emerged, *F*(5.96, 304.29) = 4.73; *p* < 0.001; *ƞ*_*p*_^*2*^ = 0.085. Univariate follow-up tests revealed that the whole sample committed more mistakes in block 1 than in blocks 2 and 3 when presented with angry, disgusted, and sad faces (all *ps* < 0.01). The mistakes in neutral expression recognition were higher in block 1 than in block 2 (*p* < 0.001), but similar to block 3 (*p* = 1.00). No significant differences in accuracy between blocks were detected for fearful and happy expressions.

**Table 1:** Mean scores and standard deviations of % of mistakes and absolute dwell time (ms) during free-, eye- and mouth-gaze blocks (*N* = 52)

	Free-gaze			Eye-gaze			Mouth-gaze		
	% Mistakes	Dwell time eyes	Dwell time mouth	% Mistakes	Dwell time eyes	Dwell time mouth	% Mistakes	Dwell time eyes	Dwell time mouth
	M (SD)	M (SD)	M (SD)	M (SD)	M (SD)	M (SD)	M (SD)	M (SD)	M (SD)
Anger	26.92	4158.60	1649.13	9.62	3800.11	1672.80	3.85	2584.11	2814.60
	(2.39)	(1567.77)	(1062.57)	(3.37)	(2346.43)	(2029.36)	(2.32)	(2330.91)	(2380.16)
Disgust	27.24	4577.51	1333.51	15.39	4050.84	1444.29	14.42	2987.83	2619.93
	(2.63)	(1949.11)	(965.31)	(3.51)	(2172.30)	(1657.81)	(3.45)	(2539.41)	(2398.60)
Fear	17.31	4607.94	1273.85	10.58	3738.40	1261.98	10.57	2687.47	2293.68
	(3.30)	(1691.32)	(911.87)	(3.17)	(2549.03)	(1481.28)	(3.46)	(2497.81)	(1916.38)
Sadness	50.00	4258.17	1334.96	28.85	4771.27	1352.56	29.81	3461.33	2163.81
	(2.90)	(1749.76)	(1196.99)	(4.83)	(2411.29)	(1439.08)	(4.81)	(2805.80)	(2222.11)
Neutral	9.94	4423.21	1239.20	2.89	4054.50	1352.21	8.65	3198.27	1907.18
	(2.21)	(1885.21)	(993.65)	(1.63)	(2671.79)	(1479.46)	(2.98)	(2986.61)	(1978.93)
Happiness	2.24	3523.92	1593.03	2.89	4191.26	1754.50	0.00	2171.10	2807.73
	(0.92)	(1652.63)	(1089.63)	(1.63)	(2371.35)	(1760.01)	(0.00)	(2311.34)	(2369.84)

### Visual Behavior

For the analyses of dwell time using the eye-tracking procedure, descriptive statistics for the whole sample in each block are shown in Table 1. Results from repeated measures ANOVA with absolute dwell time on the eyes as a within-group factor detected significant main effects of block, *F*(1.65, 83.98) = 16.39; *p* < 0.001; *ƞ*_*p*_^*2*^ = 0.24, and emotion *F*(5,255) = 5.25; *p* < 0.001; *ƞ*_*p*_^*2*^ = 0.09. A significant block*emotion interaction effect was also identified, *F*(7.45, 382.42) = 2.29; *p* = 0.024; *ƞ*_*p*_^*2*^ = 0.043. Univariate follow-up tests showed that there were no differences between blocks 1 and 2 for any emotion. Dwell time on  the eyes in block 3 was significantly lower compared to blocks 1 and 2 in angry, disgusted, fearful, neutral and happy expressions (all *ps* < 0.05). For sad expressions, dwell time to eyes in blocks 1 and 2 was similar. However, participants spent less time looking at the eyes in block 3 in comparison only to block 2 (*p* = 0.01).

When absolute dwell time on the mouth region was entered as a within-group factor, main effects of block, *F*(1.43,72.65) = 15.52; *p* < 0.001; *ƞ*_*p*_^*2*^ = 0.23, and emotion emerged, *F*(5,255) = 5.04; *p* < 0.001; *ƞ*_*p*_^*2*^ = 0.09). Univariate follow-up tests showed that dwell time on the mouth in block 1 and block 2 was similar (*p* = 1.00). However, dwell time was significantly higher in block 3 compared to both block 1 (*p* < 0.001) and block 2 (*p* = 0.002).

With regard to emotion type, the pairwise comparisons detected that dwell time on the mouth was significantly higher in angry (*p* = 0.009) and happy (*p* = 0.006) expressions when compared to neutral faces. Altogether, these findings suggest that during free-gaze, participants tended to pay attention to the eyes during facial expression presentation. When instructed to look first at the eyes or mouth, they followed the given instructions.

#### CU Traits and Emotion Recognition Accuracy

To analyze the effects of CU traits on emotion recognition accuracy, repeated-measures ANOVAs were computed with the addition of group as a between-subject factor (low vs. high based on CU or ICU and present vs absent LPE measures), using GD, INS and age as covariates. No significant main or interaction effects emerged, and no differences in the percentage of mistakes were identified between groups. Consequently, these findings suggest no associations between accuracy in emotion recognition of facial expressions and CU traits.

#### CU Traits and Visual Behavior

**Fig. 1:**
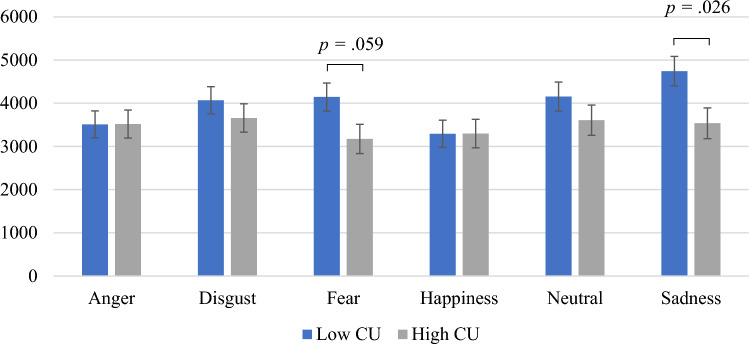
CU traits (CPTI) and emotion interaction effect on absolute dwell time (ms) on the eye region across the whole task. *CU* callous-unemotional, *CPTI* Child Problematic Traits Inventory

When using the CU scale from the CPTI to form groups, the repeated measures ANOVA predicting absolute dwell time on the eyes with CU-CPTI group as between-subject factor, and GD, INS and age as covariates, detected a two-way interaction of CU-CPTI*emotion *F*(5,235) = 2.80; *p* = 0.018; *ƞ*_*p*_^*2*^ = 0.06 (see Fig. 1). Univariate follow-up tests indicated that regardless of the block, participants in the high CU-CPTI group (*M* = 3.54; *SE* = 0.36) spent less time looking at the eyes during sad expressions, compared to those in the low CU-CPTI group (*M* = 4.75; *SE* = 0.34*; p* = 0.026*; d* = 0.69). Trend level significance was also achieved for fear expressions, indicating that those in the high CU-CPTI group (*M* = 3.17; *SE* = 0.34) fixated less time on the eyes than the low CU-CPTI group (*M* = 4.14; *SE* = 0.32*; p* = 0.059*; d* = 0.59), with a medium effect size. For the analyses of the dwell time on the mouth area, no significant main or interactive effects were identified.

For the groups formed using the ICU, ANOVA results using dwell time on the eyes or mouth as the dependent variable, with ICU group included as a between-subjects factor and GD, INS and age as covariates, indicated no significant main or interaction effects. These findings suggest that there were no differences between groups in attentional level to either the eyes or mouth area regardless of the block or emotion presented.

**Fig. 2:**
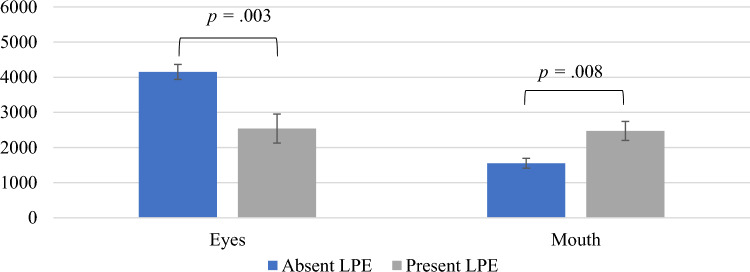
Main effects of the LPE specifier (CAPE) on absolute dwell time (ms) on the eyes and mouth regions collapsed across blocks and emotions. *LPE* limited prosocial emotions specifier, *CAPE* Clinical Assessment of Prosocial Emotions

For groups formed using the CAPE, the analysis of dwell time on the eyes with the LPE specifier as a between-subjects factor and GD, INS and age as covariates yielded a significant main effect of group, *F*(1,46) = 10.21; *p* = 0.003; *ƞ*_*p*_^*2*^ = 0.18 (see Fig. 2). Univariate follow-up tests indicated that regardless of the block or emotions, participants in the LPE group spent less time looking at the eyes (*M* = 2.54; *SE* = 0.41), compared to those with no LPE deficits (*M* = 4.15; *SE* = 0.21*; p* = .003*; d* = 1.21), with a large effect size. With regard to the dwell time on the mouth area, a significant main effect of group arose, *F*(1,46) = 7.72; *p* = 0.008; *ƞ*_*p*_^*2*^ = 0.14. Follow-up tests indicated that the dwell time on the mouth was higher in the LPE group (*M* = 2.47; *SE* = 0.27) compared to the absent LPE group (*M* = 1.55; *SE* = 0.14*; p* = 0.008*; d* = 1.05). Altogether, these findings would suggest that participants with the LPE specifier were less attentive to the eyes and more attentive to the mouth for all emotions and blocks.

## Discussion

This study was designed to examine whether youth referred for conduct problems who are elevated on CU traits show deficits in their emotion recognition accuracy and visual behavior to emotional faces. The current findings showed that: (a) low and high CU traits groups did not differ in their emotion recognition accuracy regardless of the method of assessment; (b) high CU groups paid less attention to the eyes of faces, and instead were more attentive to the mouth area; (c) the observed deficits in high CU groups were independent of the provided instructions; and (d) the results varied between assessment measures revealing that groups based on the presence of the LPE specifier using a clinical interview showed more severe attention deficits than the groups formed by behavior rating scales.

With regard to the first finding, CU traits were not related to impaired emotion recognition, contrary to our hypotheses. This finding is contrary to a significant amount of past empirical research finding this association (see [20, 32] for meta-analyses), as well as theories that propose that emotional recognition deficits can play a causal role in the development of CU traits [18, 64]. Nonetheless, there are other studies within clinical samples that also did not find difficulties in accuracy when recognizing emotions in youth elevated on CU traits [30, 34]. Instead, some authors have proposed that recognition deficits associated with CU traits may be explained by a longer processing time rather than general inaccuracy [34]. In this study, the reaction time was not registered. However, since the youth had unlimited time for answering, and it was the experimenter who registered the answers, this could have helped to increase the accuracy. Alternatively, since the whole sample committed few mistakes in general (with the exception of sadness), the results could also indicate that identifying basic emotions from static stimuli might not be challenging enough to detect difficulties in emotion recognition. The use of morphing faces, video clips, or complex emotions could increase the difficulty and ecological validity of the task, being more representative of real-life social interactions. In fact, researchers who have studied the emotion recognition skills of youth with CU traits using both static and dynamic stimuli, as well as basic and complex emotions, have found that the effects were more robust in dynamic tasks [65] and when identifying complex emotions [66]. The lack of findings related to emotional recognition may also be due to the large age range of our sample. That is, there are some findings suggesting that the emotional recognition deficits in those high on CU traits decrease with age [41, 67]. While we used age as a covariate in our analyses, our sample was not large enough to test interactions with age. Finally, another possible explanation for our lack of findings on the emotion recognition tasks could be that this study did not include a control group of children without problems, since all participants had conduct problems severe enough to warrant referral for treatment, which could also hinder detecting statistical differences between groups.

Concerning the main findings, our results supported our hypothesis that youth with high CU traits would pay less attention to the eye region of human faces. For this dependent variable, there were different outcomes between the methods used for group formation. When the groups were created based on the CAPE, the group with the LPE specifier displayed general attentional deficits to the eyes and increased attention to the mouth area, across all emotions and blocks. These findings suggest that children and adolescents with clinically significant CU traits (i.e., LPE specifier) show deficits at directing attention to emotionally salient cues (people’s eyes), consistent with previous results [25, 27, 28]. In addition, the increased attention to the mouth area is indicative of an ineffective pattern of processing emotions [25]. However, when a rating scale was used (CU-CPTI), the group with high CU traits only showed less attention to the eyes for sad faces across all blocks and a trend for less attention to the eyes for fearful faces. These results support that the deficits in processing distress stimuli may be particularly pronounced for these emotions because they are found even when forming groups in less rigorous ways that could result in more misclassifications [27, 28, 33, 68]. The stronger effects for faces depicting distress would support theories focusing on amygdala dysfunction as being a key neurocognitive process related to CU traits [8] and are consistent with findings showing different amygdala responses for children with conduct problems who are elevated on CU traits compared to those without elevations on these traits [44].

The fact that, when a clinical interview is used, the deficits are more pervasive, also has important implications. It suggests that some of the inconsistent findings from past research may have been due to the use of less rigorous methods for forming subgroups of children with conduct problems. Given that subgroups of children with conduct problems can show very different patterns of emotional deficits, misclassifications can have a significant effect on the findings and interpretation of results [12]. Misclassification can also have important implications for the design of interventions for youth with elevated CU traits that target the underlying neurocognitive mechanisms that lead to CU traits. That is, interventions can help parents learn to teach children how to make eye contact in situations that require emotional engagement from the child (e.g., discussing feelings, giving instructions...[69]). Some promising interventions have shown that it is possible to increase the eye gaze of children with elevated CU traits during parental interactions [70]. Importantly, these intervention strategies are likely to be more effective in children who show the most severe deficits documented by clinical interviews and for younger children who may be more responsive to intervention [71–74].

Another important finding is that the results showed that the whole sample followed the instructions in the eye-gaze and mouth-gaze conditions. The obtained results partially supported the proposed hypothesis based on Dadds’ studies [28, 41], since both high and low CU traits groups committed fewer mistakes when instructed to focus on the eyes of the emotional faces (eye-gaze condition) compared to the condition when such instructions were not made (the free-gaze condition). However, contrary to the work of Dadds and colleagues, the recognition accuracy of both groups between eye-gaze and mouth-gaze conditions was similar. This finding could be explained by the low number of mistakes in the whole sample, probably due to the task characteristics discussed above. With regard to visual behavior, there were no differences between groups across blocks due to a general tendency to focus less on the eyes and more on the mouth among children high on CU traits. Thus, contrary to the findings of Dadds et al. [28], our results suggested that just giving the instructions to attend to a specific area was not enough to alter the natural gaze pattern of children with elevated CU traits. As a result, interventions designed to change patterns of eye gaze may require more than just simple instruction but also may need to include reinforced practice in the skill [17].

Finally, as noted above, more generalized deficits in attention to the eye region and enhanced attention to the mouth across different emotions were found when using a clinical interview (CAPE). When using rating scales, attentional deficits to eyes were either specifically linked to sadness and, to a lesses extent, to fear (CPTI) or no attentional deficits were found at all (ICU). This finding could be due to the fact that CAPE scores in most cases (67%) also relied on interviews with children, but ratings on the CPTI or ICU did not include child report. Thus, these deficits could be more strongly related to self-report of CU traits, and this would be consistent with findings reported by Matlasz et al. [75] supporting the greater validity of self-report of CU traits across childhood and adolescence, when compared to teacher and parent report. Alternatively, the stronger findings for the CAPE could also be due to the fact that this assessment required clinicians to assess a number of parameters to ensure that the symptoms of the LPE are clinically significant (i.e., that they were impairing, persistent, and pervasive). This possibility would be in line with previous suggestions that CU traits might be particularly difficult to assess with behavior rating measures [76].

Importantly, past tests of the validity of the CAPE have largely relied on validating the measure as a way of detecting subgroups of youth with conduct problems with the LPE specifier who show more severe behavior problems [56, 57, 77]. Our findings support its validity for designating a group of youth with conduct problems who show more severe emotional deficits as well. Our findings are consistent with a study by Neo et al. [58] who reported that young children (ages 2 to 8 years) who met criteria for the LPE specifier according to the CAPE showed less accuracy in recognizing emotions than children with behavior problems who were not elevated on the CAPE. The findings by Neo and colleagues also support our contention that our lack of findings related to emotional recognition deficits may have been due to the older age of our sample.

All of these interpretations need to be made in the context of several study limitations. Our sample was relatively small, and the statistical power did not allow us to detect small effects, although our sample size was similar to other studies using clinical or high-risk samples (e.g., [25, 27]). Also, a primary purpose of our study was to compare methods for creating subgroups of children and adolescents with conduct problems who differed in their level of CU traits. We specifically wanted to compare the validity of a clinical approach to making this dichotomous diagnosis, compared to approaches using elevations on behavior rating scales. Comparing more extreme groups would have been ideal, but our sample size was not sufficient for such stratification. However, given that our sample was all children referred for conduct problems, we feel that a median split could be justified but also a limitation to consider when interpreting our results. Regarding the laboratory task, facial stimuli were selected from different sets and were slightly different in size, which led to a wide definition of areas of interest. In addition, our models for the facial expression used only male models, and it is not clear if our findings would have been different with the use of female or child models. Moreover, the sample was composed only of boys with heterogeneous clinical profiles; as a result, these findings cannot be generalized to girls or community samples.

In conclusion, our study found that CU traits were unrelated to emotion recognition accuracy of basic emotions from static emotional facial stimuli in a sample of youth with conduct problems spanning a large age range. CU traits instead were associated with less attention to the eye region and more attention to the mouth area. These results were somewhat dependent on the way that CU traits were assessed. A structured clinical assessment of CU traits identified a group that showed more severe (i.e., see Cohen’s d effect sizes) and pervasive deficits across emotions, whereas behavior rating scales detected smaller effects and only deficits in response to sad expressions. Thus, this study helps to clarify possible reasons for inconsistent findings in past research, contributes to knowledge on the mechanisms that may underlie CU traits, and provides evidence to help guide optimal ways to assess CU traits and to guide the development of novel interventions for treating this group of youth with conduct problems who have shown only a limited response to past approaches to treatment [5].

## Summary

Deficits in emotion recognition and reduced attention to others’ eyes seem relevant to the development of CU traits in children and adolescents. This study expands knowledge about the associations between emotion recognition, visual behavior, and CU traits in a sample of 52 boys (aged 6-14) referred to mental health services for conduct problems. CU traits were assessed by multiple informants (parents, teachers, and boys), and the results were compared between high and low CU traits subgroups created using different methods, including behavior rating scales (CPTI, ICU) and a clinical interview (CAPE). First, the findings revealed that high CU traits did not differ from low CU traits subgroups in emotion recognition accuracy, regardless of the method used. Second, boys with high CU traits showed reduced attention to the eyes and increased attention to the mouth area. These results highlight abnormalities in emotional processing in children with high CU traits, suggesting the need for tailored assessments and interventions. Finally, when the different methods were compared, more severe and pervasive deficits in attentional patterns were detected using the clinical interview (CAPE). Future studies should include multiple informants and methods in the assessment of CU traits.

## Data Availability

The data that support the findings of this study are available from the corresponding author upon reasonable request.

## Footnotes

1 For reasons unrelated to the study, the first rater was not able to provide the CAPE scores for all participants. To overcome this issue, we used the second rater's scores due to a greater number of available data (*n* = 51) and substantial interrater reliability.

2 Within one week after the family provided consent, we contacted the school’s director to get permission to contact the teacher. Once the school agreed, we contacted the teacher, explained the procedure, and obtained their consent. After that, we conducted the CAPE interview with the teacher, usually within a month. However, in some cases this timing was more flexible to ensure maximum participation of the teachers, particularly during the COVID-19 lockdown when the schools were completely closed.
